# Geographical variation in radiological services: a nationwide survey

**DOI:** 10.1186/1472-6963-7-21

**Published:** 2007-02-15

**Authors:** Kristin Bakke Lysdahl, Ingelin Børretzen

**Affiliations:** 1 Radiography Programme, Faculty of Health Sciences, Oslo University College, P.O. Box 4, St. Olavs plass, NO-0130 Oslo/Section for Medical Ethics, Faculty of Medicine, University of Oslo, P.O. Box 1130, Blindern, NO-0318 Oslo, Norway; 2 Norwegian Radiation Protection Authority, P.O. Box 55, NO-1332 Østeraas, Norway

## Abstract

**Background:**

Geographical variation in health care services challenges the basic principle of fair allocation of health care resources. This study aimed to investigate geographical variation in the use of X-ray, CT, MRI and Ultrasound examinations in Norway, the contribution from public and private institutions, and the impact of accessibility and socioeconomic factors on variation in examination rates.

**Methods:**

A nationwide survey of activity in all radiological institutions for the year 2002 was used to compare the rates per thousand of examinations in the counties. The data format was files/printouts where the examinations were recorded according to a code system.

**Results:**

Overall rates per thousand of radiological examinations varied by a factor of 2.4. The use of MRI varied from 170 to 2, and CT from 216 to 56 examinations per 1000 inhabitants. Single MRI examinations (knee, cervical spine and head/brain) ranged high in variation, as did certain other spine examinations. For examination of specific organs, the counties' use of one modality was positively correlated with the use of other modalities. Private institutions accounted for 28% of all examinations, and tended towards performing a higher proportion of single examinations with high variability. Indicators of accessibility correlated positively to variation in examination rates, partly due to the figures from the county of Oslo. Correlations between examination rates and socioeconomic factors were also highly influenced by the figures from this county.

**Conclusion:**

The counties use of radiological services varied substantially, especially CT and MRI examinations. A likely cause of the variation is differences in accessibility. The coexistence of public and private institutions may be a source of variability, along with socioeconomic factors. The findings represent a challenge to the objective of equality in access to health care services, and indicate a potential for better allocation of overall health care resources.

**Previous publication:**

The data applied in this article was originally published in Norwegian in: Børretzen I, Lysdahl KB, Olerud HM: Radiologi i Noreg – undersøkingsfrekvens per 2002, tidstrendar, geografisk variasjon og befolkningsdose. StrålevernRapport 2006:6. Østerås: The Norwegian Radiation Protection Authority. The Norwegian Radiation Protection Authority has given the authors permission to republish the data.

## Background

Geographical variation in use of radiology has been documented in the United States [1-3], in Europe [4], and in the Nordic countries, where a factor of 1.8 in variation between the countries was reported [5]. When areas differ with respect to available economic resources, health care policies, referral systems, and reimbursement policies, then geographical variation can be expected. In Norway the health care system is predominantly public, and the National Insurance Scheme covers the vast majority of radiology services (including private radiology services), that is when patients obtain a referral from their physician (GP or specialist).

In an earlier study differences in use rates of radiology were observed between urban and rural areas in Norway, but the differences were not quantified [6]. To determine the presence and magnitude of variability of radiology is important with respect to the question of equal access to health care, which is a declared objective in Norwegian health policy (as in most other countries), and stated in The Patients' Rights Act [7]. Moreover, such data is valuable with respect to the question of allocation of overall health care resources, especially as radiology is a costly discipline, and expenditure on radiology is steadily increasing [8]. Although the question of the correct or reasonable level of utilization cannot be answered through small area variation analysis, a significant variation in otherwise homogeneous areas may indicate that use of radiological services, at least in some areas, is not optimal [2,5]; that is, that underuse or overuse occurs.

The aim of this study was to determine the use rates of radiology in Norwegian counties. Variation in overall and modality specific examination rates, i.e. X-ray (XR), Computed Tomography (CT), Magnetic Resonance Imaging (MRI) and Ultrasound (US), was analysed, as well as variability in clustered and single examinations. (In this study, XR includes conventional radiography, fluoroscopy, mammography, angiography and interventional procedures). In addition, the following hypotheses were investigated: a) counties with low use of one modality for examination of specific organs (locations) have correspondingly high use of other modalities, b) private institutions contribute to geographical variation, and geographical variation in examination rates is influenced by c) different aspects of accessibility and d) socioeconomic factors.

## Methods

The Norwegian Radiation Protection Authority (NRPA) has conducted a nationwide survey to determine the frequency of radiological examinations during the year 2002. All medical radio-diagnostic procedures are included except dental examinations, nuclear medicine, bone densitometry and radiology in chiropractic settings and primary health care services. Activity data were received from all addressed entities (referred to as institutions in the following); 71 public and 9 private hospitals, 25 mammography screening laboratories and 25 private radiology enterprises. According to NRPA's registers these include all relevant institutions in 2002, hence the results are based on a complete count of the included examinations (not a sample survey).

The requested data format was files or printout from the Radiology Information Systems (RIS), where examinations are recorded according to the Norwegian Radiological Codes system (NORAKO) [9]. This system provides detailed information on the organ/organ system/anatomical region examined (location code), the modality used (modality code), the type of procedure to characterise/specify the examination (procedure code). Codes for right, left, or bilateral examination (side code) and codes for additional information (such as anaesthesia) are also included.

### Data preparation

Of the 130 institutions, 13 did not report in the requested format. For 5 of these, the examinations were coded manually on the basis of received descriptive texts. For 8 (small) institutions that reported on examination group level (e.g. number of skeleton X-rays), average distributions for similar institutions were used to estimate the distribution of localization codes within each group.

The main adjustments carried out concern estimation of number of examinations from the number of codes. Since the codes are primarily used for reimbursement purposes, activities reflecting resources utilized in the examinations need to be specified. Consequently, one examination can generate more than one code. In general, codes were deleted when they did not represent an examination; either as stated explicitly by the institution (e.g. reinterpretation of radiographs) or by the user manual prepared by the Norwegian College of Radiology [9]. Procedure codes for additional series were deleted, except for six institutions where coding had clearly not been carried out in accordance with the manual. This adjustment has reduced the number of CT and angiography codes by 7.7% and 8.7% respectively, while 40% of the MRI codes were deleted. Moreover, in CT examinations 7.8% of the intravenous contrast (IV) codes were deleted to eliminate double coding (of examinations both with and without IV). This adjustment was estimated based on a previous survey of CT examination techniques [10]. In X-ray examinations (other than angiography) the number of codes was assumed to be equal to the actual number of examinations, due to a low probability of any kind of double coding. A more detailed description of the method is to be found in an extended report [11].

County was the entity used in the geographical variation analyses, based on the assumption that institutions mainly serve inhabitants in their own county. For mammography screening this was not the case; three screening laboratories provided services in two counties each. Consequently, these data were distributed according to the number of inhabitants in the respective counties. Moreover, for two large hospitals situated in Oslo (the Norwegian Radium Hospital Comprehensive Cancer Centre and Rikshospitalet University Hospital) the majority of the patients reside outside Oslo (close to 90%). Detailed information on patients' residential county and the corresponding number of examinations per modality was provided, and formed the basis for distributing these data.

### Indicators of accessibility and socioeconomic status

The reported data on number of examinations are not linked to individual patients, hence correlations between examination rates and variables of accessibility and socioeconomic factors are provided on an aggregated level. Information on settlement characteristics (population density, percentage of urban settlements) and number of radiographers employed in the counties were obtained from Statistics Norway [12], while number of working radiologist in each county was obtained from The Norwegian Medical Association (on personal request). In addition the proportion of the counties population living in municipalities with general radiological services was calculated. This calculation was based on number of inhabitants in each municipality (the 19 counties are divided in to 434 smaller municipalities) and information of location of general radiological services (delimited by excluding mammography screening facilities and specialised rehabilitation and heart disease hospitals). This measure was applied to assess the impact of proximity, i.e. whether a high percentage of a county's population living in a municipality where a radiological provider exists could explain high examination rates. Socioeconomic information (average gross income for persons 17 years of age and over, and number of persons with tertiary or postgraduate level of education per 1000 inhabitants) was also obtained from Statistics Norway. The linked table displays figures of settlement characteristic, radiological resources and socioeconomic factors in the counties (see Additional file 1).

### Statistics

Geographical variation is presented as rates (number of examination per 1000 inhabitants) in each county, high/low ratio for rates (an easily comprehensible measure), and the more accurate variation measure coefficient of variation (COV, which is defined as standard deviation relative to mean rate value). Pearson's r describes correlations. Statistical significance analyses is performed even though the results are based on complete count, seeing the year 2002 data as a sample of the true values that varies randomly across years.

## Results

The overall reported rate per thousand of radiological examinations in Norwegian counties in 2002 varies by a factor of 2.4. The rate was highest in Oslo (the capital and the most densely populated county) and lowest in Finnmark (the most distant and sparsely populated county): 1487 and 613 examinations per 1000 inhabitants respectively. The linked map displays the location of the counties (see Additional file 2).

Geographical variation in the use of different modalities is shown in Table 1, in terms of examination rates. For every modality the rates were highest in the county of Oslo. The national mean rate in use of overall Computed Tomography (CT) was 104 examinations per 1000 inhabitants; the rate ranged from 216 in Oslo to 56 in Oppland (a mainly rural county in the eastern part of the country). This difference represents nearly a fourfold variation. The variation in use of Magnetic Resonance Imaging (MRI) was even greater. The national average use rate of MRI imaging was 61 examinations per 1000 inhabitants; the use rate ranged from 170 in Oslo to 2 in Finnmark. The variation in use of X-ray (XR) and Ultrasound (US) was less: The ratios of highest (Oslo) and lowest rates (Finnmark and Nord-Trøndelag) were 2.0 and 2.9 respectively. The coefficients of variation (COV) were 69% for MRI, 36% for CT, 27% for US and 2% for XR, i.e. the highest variation was found for the most recent and advanced technologies.

**Table 1:** Number of examinations per 1000 inhabitants^1 ^according to modality and county in 2002, with high/low ratio and coefficient of variation (COV)^2^

**County**	**XR**	**CT**	**MRI**	**US**	**Total**
Oslo	921	216	170	180	1 487
Telemark	823	97	80	135	1 134
Vest-Agder	754	114	98	101	1 067
Troms	778	108	65	113	1 064
Østfold	691	122	84	139	1 036
Sør-Trøndelag	671	121	57	98	947
Buskerud	656	80	58	131	925
Vestfold	612	86	72	111	882
Nordland	576	106	60	94	836
Rogaland	589	89	34	95	807
Hordaland	573	95	25	88	781
Hedmark	565	83	12	102	761
Møre og Romsdal	544	72	29	103	749
Akershus	522	77	40	69	707
Nord-Trøndelag	533	78	33	62	706
Sogn og Fjordane	505	72	37	86	700
Aust-Agder	484	61	34	71	649
Oppland	484	56	26	82	648
Finnmark	459	72	2	80	613
					
High/low ratio	2.0	3.9	83.7	2.9	2.4
COV	20	36	69	27	24

^1 ^Total population in Norway 2002: 4 552 252

^2 ^COV is standard deviation relative to mean rate value within counties

When examinations were clustered according to the region of the body (irrespective of the modality used), the highest variability was seen for spine examinations (COV: 39%, high/low ratio: 4.3). The least variation was found for 'chest' examinations (COV: 18%, high/low ratio 1.9). Medium variation was found for 'pelvis, urinary tract and genitalia', 'head', 'mammae', 'abdominal and gastrointestinal' 'extremities' (COV between 34% and 24%, high/low ratio between 3.9 and 2.2).

Geographical variation for single examinations is shown in Table 2, the 30 most frequent (of a total of 370) are included. Altogether these include 80% of all examinations reported. The three MRI examinations that were included (knee, cervical spine and head/brain) all ranged high in variation (COV: 103%, 79% and 47%). It can also be noticed that different kinds of spine examinations ranged high. XR thorax was the one that varied the least (COV: 16%). However, the rate of this examination was twice as high in the county of Telemark as in Oppland (160 vs. 82 examinations per 1000 inhabitants respectively), two counties with similar demographic characteristics.

**Table 2:** Geographical variation in number of single examinations^1 ^per 1000 inhabitants, in the counties with highest and lowest values, high/low ratio and COV within counties

	**Highest**	**Lowest**	**High/low ratio**	**COV (%)**
MRI Knee	37	0.1	304.1	103
US Pelvis	17	0.2	86.7	79
MRI Cervical spine	21	0.2	139.0	79
XR Lumbar spine	29	2.0	14.4	78
US Upper urinary tract	22	2.8	8.0	57
CT Lumbar spine	34	3.5	9.6	51
US Mammae	21	2.5	8.3	50
MRI Head/brain	26	0.8	32.4	47
CT Pelvis	22	4.4	5.0	45
XR Lumbar spine with sacrum	39	7.0	5.5	44
XR Cervical spine	43	9.6	4.5	40
XR Pelvis	54	13.9	3.9	37
CT Abdomen	24	7.5	3.2	34
CT Thorax	20	6.1	3.3	33
XR Thorax, front	71	14.3	4.9	32
XR Shoulder	46	13.9	3.3	31
XR Thoracic spine	14	4.9	2.9	31
US Abdomen	39	9.7	4.0	30
XR Urinary tract/urography	18	7.2	2.5	27
XR Mammography	105	33.2	3.2	27
XR Knee	57	21.5	2.7	26
CT Head/brain	52	19.7	2.7	25
XR Foot	38	16.2	2.3	25
XR Ankle	36	16.6	2.2	23
XR Hip	64	28.7	2.2	23
XR Lower leg	13	5.3	2.4	23
XR Hand/fingers	41	13.7	3.0	22
XR Elbow	13	5.7	2.3	22
XR Wrist	33	12.2	2.7	22
XR Thorax, two projections	160	82.2	1.9	16

^1 ^The 30 most frequent examinations are included

### Correlations between different modalities

**Figure 1:**
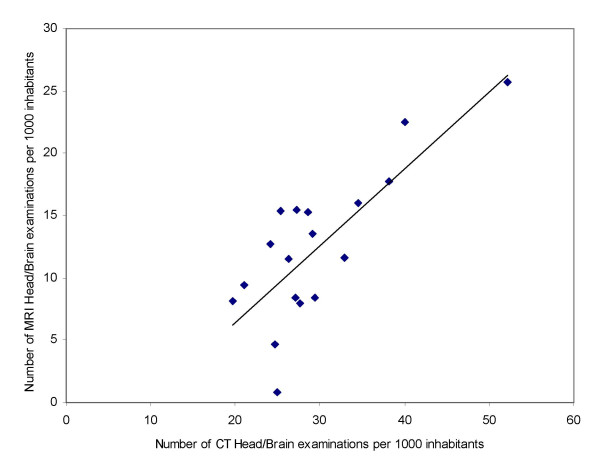
**Correlation between rates per thousand of CT and MRI Head/Brain examinations in Norwegian counties**. Regression line is displayed.

One could expect a tendency that a low use rate of one modality in examinations of specific organs (locations) corresponded with a high use rate of alternative modalities. However, the results showed a relatively high positive correlation between the uses of different modalities for specific organs of examination, as shown in Table 3. This was most pronounced for CT and MRI examinations of head/brain, CT and MRI of pelvis, XR and CT of thorax, XR and MRI as well as CT and MRI of cervical spine and finally XR and MRI of knee (all Pearson's r > 0.63 and significant at a 0.01 level, 2-tailed). Several other correlations are significant at a 0.05 level. Of the 24 correlations 23 was positive, the binomial distribution probability of this is lower than 0.000. The correlation for head/brain examinations is illustrated in Figure 1.

**Table 3:** Correlations (Pearson's r) between examination rates according to modality^1 ^within counties

**Examination**	**XR and CT**	**XR and MRI**	**XR and US**	**CT and MRI**	**CT and US**	**MRI and US**
Head/brain	-	-	-	0.78**	-	-
Cervical spine	0.35	0.73**	-	0.68**	-	-
Lumbar spine	-0.06	0.38	-	0.56*	-	-
Thorax	0.75**	-	0.11	-	0.15	-
Abdomen	0.51*	0.35	0.28	0.31	0.21	0.22
Urinary tract	-	-	0.51*	-	-	-
Pelvis	0.63**	0.55*	0.39	0.75**	0.52*	0.62**
Knee	-	0.70**	-	-	-	-

^1 ^Correlations are only displayed if both modalities are frequently used for the examination in question, i.e. more than 1 per 1000 inhabitants nationally

* p < .05, ** p < .01

### Contribution from private and public institutions

**Figure 2:**
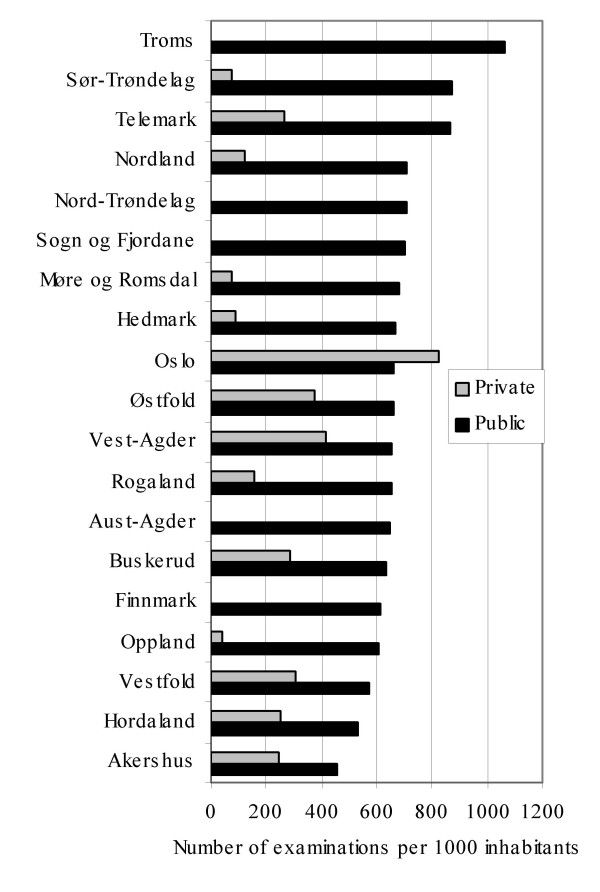
Rates per thousand of all radiological examinations in Norwegian counties, performed in public and private institutions.

Figure 2 illustrates how public and private institutions contributed to examination rates per thousand. The private institutions mainly contribute to examination rates in counties in the central south eastern part of Norway, especially in Oslo. As examinations performed in private institutions accounted for only 28% of all examinations the geographical variations was mainly caused by public institutions. However, in single examinations the contribution from private institutions differed substantially and was generally higher in examinations with high variability. Private institutions performed e.g. 78% of MRI knee, 57% of CT lumbar spine and 55% of MRI cervical spine examinations. The correlation between the variations (COV) of the 30 most frequent single examinations (the same examinations as displayed in table 2) and the proportion of these examinations performed by private institutions, Pearson's r was 0.72 (significant at a 0.01 level, 2-tailed).

### The impact of accessibility

**Figure 3:**
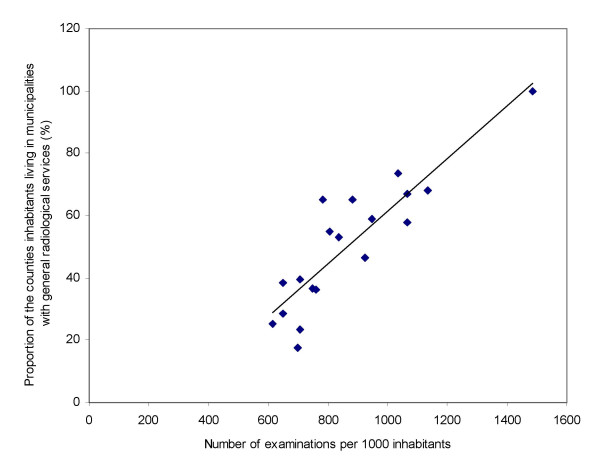
**Correlation between the proportion of the counties' population living in municipalities with general radiological services and examination rates per thousand inhabitants**. Regression line is displayed.

The correlation (Pearson's r) between examination rates per thousand and the proportion of the counties population living in municipalities with general radiological services was 0.88 (illustrated in figure 3), i.e. accessibility (indicating proximity to radiological services) statistically explains 78% of the variance of examination rates. The population density's (population per km^2^) correlation to examination rates was 0.69 and similarly the proportion of urbane settlement 0.63. The correlations between examination rates and number of radiologist and radiographers (measures of accessibility in the shape of available radiological resources) were 0.71 and 0.66 respectively. All correlations were significant at a 0.01 level (2-tailed). However, as settlement characteristics and radiological resources in Oslo seem to be substantially different from the other counties, correlation was also performed without Oslo. This diminishes all the correlations; only slightly for the proportion of the counties population living in municipalities with general radiological services (to 0.82, still significant at a 0.01 level), more markedly for urban settlement (0.41), even more for number of radiologists and radiographers (to 0.23 and 0.15), while the correlation for population density vanished (0.09). None of the 4 last variables correlations were statistically significant.

### The impact of socioeconomic factors

Two socioeconomic variables were used in this study; the number of persons with tertiary or postgraduate level of education per thousand inhabitants and the average gross income. The educational level variable correlated positively to overall examination rates (Pearson's r = 0.59, p = .008), while the income correlation was not significant (Pearson's r = 0.45, p = .056). Again the influence of Oslo was striking: the correlations vanished when the county of Oslo was not included in the analyses (r = 0.05 and 0.008 respectively). In Oslo the gross income level and the number of persons with highest level of education was a factor 1.3 and 1.8 above mean values in the counties respectively.

## Discussion

The geographical variation in use of radiology was significant, not least as Norway traditionally is considered to be an egalitarian society where great emphasis has been placed on equal access to health care services. Most studies of variation rates in radiology are limited to single examinations and/or limited to particular populations. Hence, it is difficult to assess the level of overall variation found in our study compared to other studies. However, our study is very much in line with results from studies of geographical variations with regard to particular modalities, such as CT and MRI [13].

### Validity of the data

The chosen data format was considered appropriate, as almost all institutions used the same coding system (NORAKO), it was convenient for the institutions and secured both a complete response rate and a uniform format. Nevertheless, some methodological considerations need to be discussed. The main challenge concerns estimation of the number of examinations from the number of codes. After deletion of codes that only described details about how the examinations were performed, we regard the number of examinations as almost correct. The exception is angiography/interventional procedures (and to a small extent ultrasound examinations) where data were not adjusted for the use of several location codes within a single examination. This means that the total rates per thousand was overestimated. However, the possible effect on geographical variation is small since angiography/interventional procedures accounted for only 2% of all XR examinations. If the estimation deviates in any other way from the real number of examinations, this would have a minor influence on geographical variation, as all institutions were treated in an equal manner. It could be argued that this presupposes that all institutions used the coding system in exactly the same way. When coding practice was scrutinised some differences between the institutions were discovered, possibly due to somewhat ambiguous guidelines. However, there were no systematic differences between the counties.

### Migration as a cause of geographical variation

One obvious challenge to validity is patient migration (from one county to another). For two large hospitals (both situated in Oslo) examinations were distributed according to patients' home county. Nevertheless, patient migration may take place in other institutions and be a challenge to validity. Factors reflecting different aspects of accessibility could influence migration, e.g. that the requested examination is not available in the patient's home county, or that waiting times or travelling distances are shorter in a neighbouring county. This effect certainly explains some of the differences between Oslo, and the surrounding county of Akershus. However, the merged rate per thousand for these two counties was still high. In many other parts of the country the travel distances are long and inconvenient. Exact information on patient migration in radiology is sparse, but data from one private institution in each of three counties showed that 85%, 95% and 98% of the patients lived in the county where the institution was located [14]. Indirect information is given by the fact that the number of patients who choose to be treated (in general, not limited to radiology) outside their local hospital in 2002 was small [15]. Free choice of hospital was implemented in Norway in 2001. All in all the problem of migration of patients may have caused that the variations found in this study are slightly overestimated.

### Variation caused by differences in accessibility

Availability is a well known factor for explaining variation in utilization rates for radiology [16,17], and probably contributes to the high variability of MRI and CT examinations and the strikingly high rates in Oslo (although burdened with some methodological uncertainties) found in this study. The county of Oslo is atypical, since it comprises the city and its suburbs; a highly urbanized area, with short distances to services, with large university hospitals and high availability of new high-technology imaging facilities. All these factors are associated with high use of radiology [1,17]. The radiological institutions in the counties differ in relation to size, the amount of CT and MRI equipment, and maybe also in practice pattern. Aroua [4] found that the rate of chest radiography (a simple examination available "everywhere") was almost three times higher in university hospitals than in small hospitals in Switzerland. Our study also reveals geographical variation in this "low-tech" examination. The acquisition of new technology – which occurs first in larger urban institutions – may lead to additional examinations if (as implied by Olsson [5]) willingness to give up outdated methods varies. This mechanism corresponds well with the correlation that was found between utilization of different modalities for specific organs (locations).

The special position of Oslo was confirmed by the analyses of correlation between different aspects of accessibility and overall examination rates which were markedly influenced by extreme values in this county. It seems somewhat surprising that radiological resources (number of radiologists and radiographers) was not stronger related to examination rates in the other counties. The correlation between examination rates and the proportion of the counties population living in municipalities with radiological services was high even without Oslo's contribution. On this variable the eight counties with lowest score corresponded to the eight with lowest examination rates. In the opposite end of the scale was Østfold and Telemark, in these counties a large proportion of the population lived in municipalities with radiological services, and had correspondingly high examination rates. An interesting difference in examination rates (75%) was found between Telemark and Oppland, two counties with similar population size and radiological resources. Both the population density and the proportion of urban settlement were lower in Oppland than in Telemark, but most noteworthy 68% of the population in Telemark lived in municipalities with radiological services compared with 28% in Oppland. These findings support the importance of proximity to services as explaining factor. One possible interpretation is that in areas with poor access patients (and their GPs) regard the usefulness of the examination as small in relation to the inconvenience (travelling etc) it causes. In this case the necessity of the examination cannot be perceived to be pressing. This interpretation is reasonable in the light of the kind of examinations that vary the most. For example, in cases of knee, shoulder or back pain, which often resolves with time and which does not often represents a severe or life-threatening disease, a "wait and see" approach might be more common if access to radiology is poor.

### Other sources of variability

Other frequently stated explanatory factors are socioeconomic differences in the populations [1,16-18] and economic factors [19]. In this study the educational and income level was found to have little impact on examination rates, but these results must be interpreted with caution, as the variations in socioeconomic factors are considered to be higher within than between counties. However, in Oslo the level of education and average income was clearly above mean values in the counties. People with higher socioeconomic status and higher education are presumed to use medical care more readily and to demand more elective services. Ordering an imaging test may be a response to high patient expectation, especially when combined with constrained time resources [20]. In a Norwegian survey, about 50% of the physicians occasionally or often gave priority to patients' wishes over their own medical judgement [21]. Hence, patient pressure is a major factor influencing GPs' referral behaviour. For example, Morgan et al [22] found that patient pressure was the main factor or a significant factor in 46% of knee radiograph referrals. Closely linked to this are two other factors that influence referral decisions: professional uncertainty [1,23] and medico-legal considerations. Kristiansen et al [24] found that the likelihood of having experienced negative reactions from patients was higher among doctors in central Norway.

Economic incentives can affect both the referring and performing physician/institution. The density of GPs is higher in urban areas. How this affects referral patterns when remuneration is based on a per capita component and a fee-for-service component is ambiguous. GPs may compensate for shortage of patients by providing more services to each patient [25]. On the other hand, they might act more in compliance with patients' wishes for radiology, to keep patients on their lists.

Some GPs report that they refer patients to private radiological institutions when the purpose is mostly to satisfy patients' wishes, while in cases of high probability of positive findings they refer to the hospital [23]. They assume that they would have referred fewer patients if private institutions had not been available. This may be because hospitals practise referral guidelines more strictly than private practices, as indicated (in general) by Iversen and Lurås [25]. The finding that the proportion of contribution from private institutions was correlated to the variability in single examinations is interesting in this context. Besides, the kind of examinations for which both the contribution from private institutions and the variability was high fits well into this picture: examinations for which justification is controversial, e.g. knee [22] and spine [26,27], and for which serious disease is seldom revealed. Private radiological institutions are located in the large towns, 1/3 in the city of Oslo. When both private and pubic sectors are established in the same region, it is crucial for the overall radiological activity in the region whether they compete or complement each other. If this is the case is an open question.

The fact that high rates of use do not necessarily improve patient outcome [2,28], raises the question of whether geographical variation in radiological services reflects inappropriate use. Geographical variation in user rates for medical services does not determine the appropriateness of the services [29], in order to determine what constitutes overuse and underuse, data on variation must be linked to information on indications, clinical outcome, risks and costs. Analyses of clinical outcome were far beyond the scope of this study. Neither was it possible to perform quantitative analyses of variation in morbidity as explaining factor for variation in examination rates due to lack of available statistics. The nationwide health survey available are either limited to age groups or specific diagnoses that can not be easily linked to use of radiological services (e.g. cardiovascular disease and diabetes) or they are not representative on the county level. Such analyses could indirectly have illuminated the question of appropriateness of the use of radiological services in the counties. According to Leape et al inappropriate use occurs in low-use areas as well as in high-use areas [30]. Even though, in industrialised countries today, we cannot ignore the problem of patients who are referred too late or not at all [3,29], the problem of unnecessary examinations appears to be more pertinent [22,26,31,32]. Indications of supplementary examinations found in this study, that high use of one modality does not correspond with low use of an alternative modality for specific organs (locations), support the assumption that overuse exists in high-use areas. The lack of a substitution effect of one type of imaging for another has also been found in other studies, i.e. areas with higher rates of one type of imaging also had higher rates of another type [2]. The question of possible overtesting is crucial to address if we want to avoid or to minimise rationing of care to sick patients [33], as radiology is a costly enterprise [32], and overall health care resources are limited. Neglect of this issue leads to a risk of unjust diversion of resources from sick people to people who are worried but healthy.

## Conclusion

There was substantial geographical variation in use rates of radiology in Norway, especially for MRI and CT examinations. Accessibility seems to be a plausible explanation, besides socioeconomic factors and the contribution from private institutions may play a part. The study was not designed to verify/disprove overuse or underuse of radiology. However, characteristics of examinations with high variability and indications of supplementary examinations mean that overuse cannot be ruled out, but neither can underuse. We believe that the findings reveal a potential for more equal access to radiological services and for better allocation of overall health care resources.

## Competing interests

The author(s) declare that they have no competing interests.

## Authors' contributions

IB collected the data. KBL analysed the data and drafted the manuscript. IB and KBL revised and finally approved the manuscript.

## Pre-publication history

The pre-publication history for this paper can be accessed here:



## Supplementary Material

Additional File 1 **Table of settlement characteristics, radiological resources and socioeconomic factors in the counties**. The additional file displays the figures of settlement characteristics, radiological resources and socioeconomic factors in the counties that were used in the analyses. The data source is Statistics Norway [12] except from the number of working radiologists which was obtained from The Norwegian Medical Association (on personal request). Click here for file

Additional File 2 **Map of Norwegian counties**. The additional file is a map that displays the location of Norwegian counties. Click here for file
