# Supplementary figures and images for: Geographical variation in radiological services: a nationwide survey
Source: BMC Health Serv Res. 2007 Feb 15;7:21. doi: 10.1186/1472-6963-7-21 (PMC1805434; doi:10.1186/1472-6963-7-21)

## Slide 1
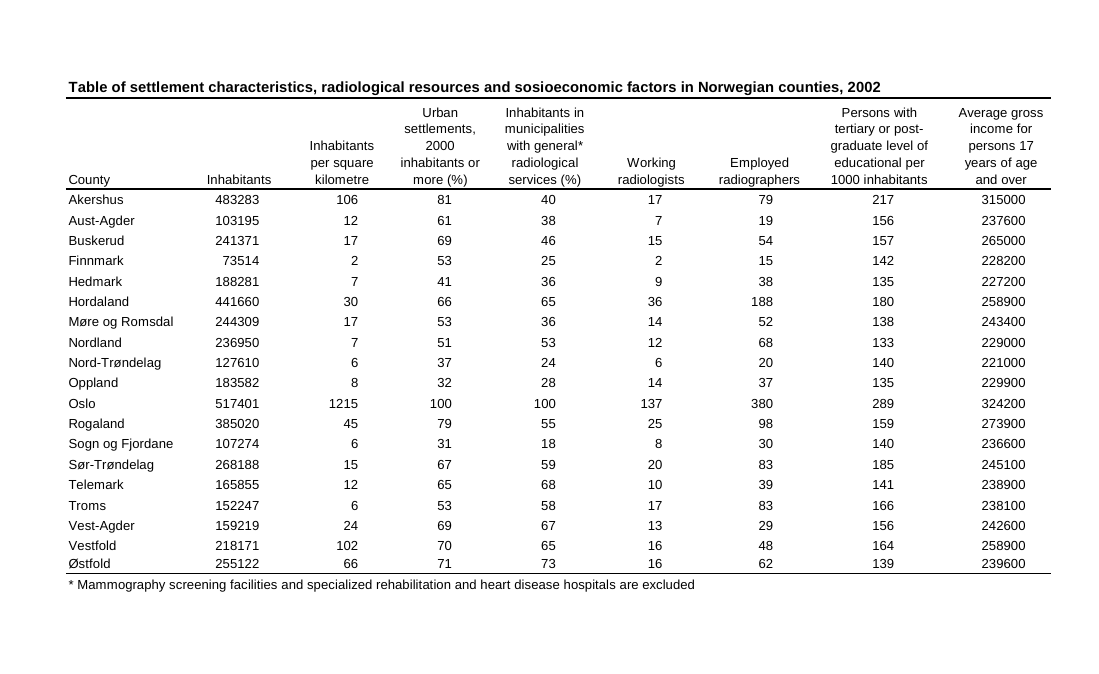

Supplement: Additional File 1 — Table of settlement characteristics, radiological resources and socioeconomic factors in the counties. The additional file displays the figures of settlement characteristics, radiological resources and socioeconomic factors in the counties that were used in the analyses. The data source is Statistics Norway [12] except from the number of working radiologists which was obtained from The Norwegian Medical Association (on personal request). [file 1472-6963-7-21-S1.ppt]

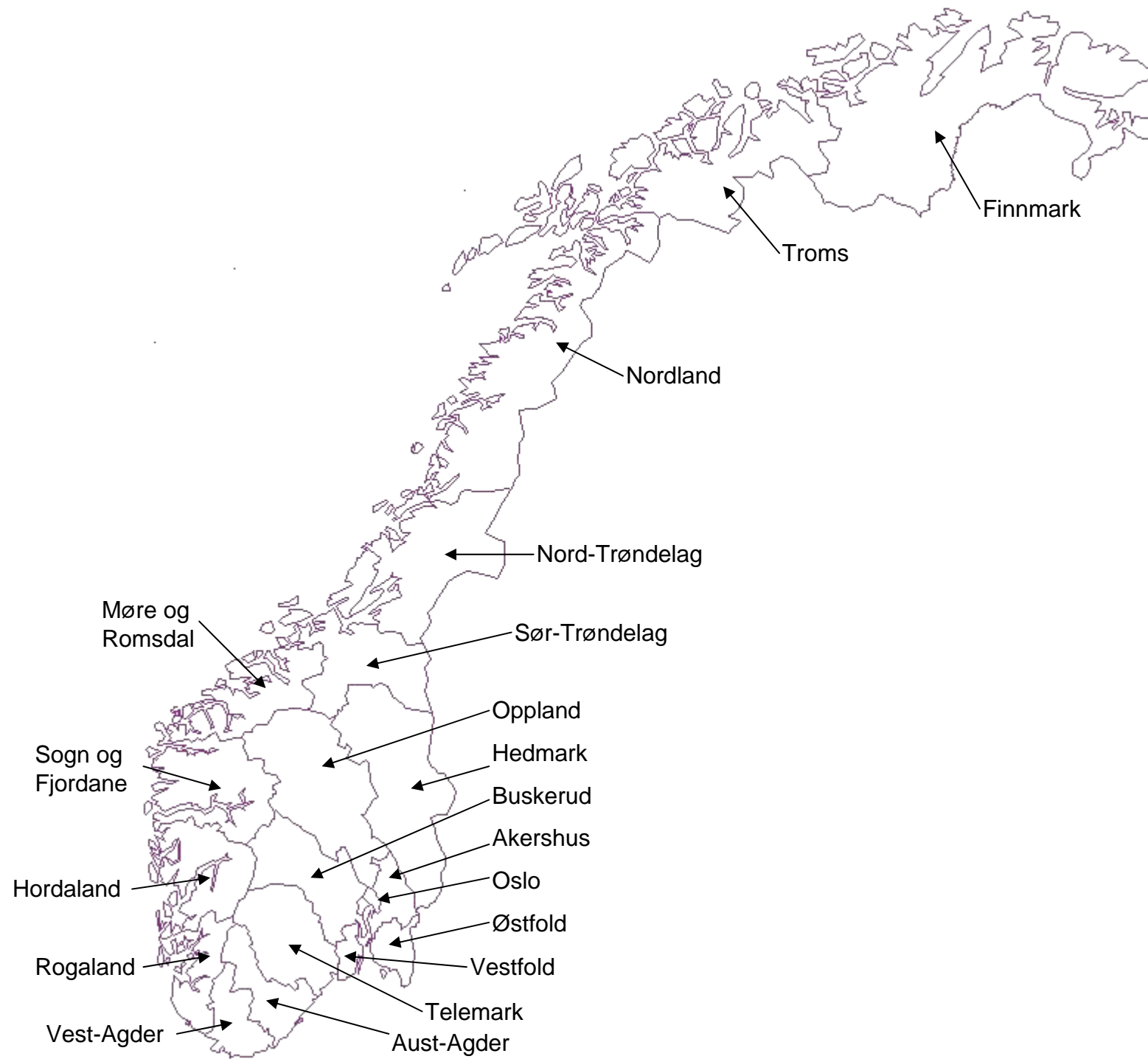

Supplement: Additional File 2 — Map of Norwegian counties. The additional file is a map that displays the location of Norwegian counties. [file 1472-6963-7-21-S2.pdf]
